# Recent advances of antioxidant low-dimensional carbon materials for biomedical applications

**DOI:** 10.3389/fbioe.2023.1121477

**Published:** 2023-01-20

**Authors:** Nan Tang, Zhen Ding, Jin Zhang, Yanting Cai, Xingfu Bao

**Affiliations:** ^1^ Department of Orthodontics, Jilin Provincial Key Laboratory of Tooth Development and Bone Remodeling, School and Hospital of Stomatology, Jilin University, Changchun, China; ^2^ Beijing Advanced Innovation Center for Soft Matter Science and Engineering, College of Life Science and Technology, Beijing University of Chemical Technology, Beijing, China

**Keywords:** low-dimensional materials, carbon, nanotheranostics, biomedical applications, nanomaterials

## Abstract

As the primary cause of many tissue damage and diseases, reactive oxygen species (ROS) and reactive nitrogen species (RNS) are well known to be extremely harmful to a variety of biological components in cells including lipids, proteins and DNA. Numerous antioxidative nanomaterials have been artificially designed and rationally synthesized to protect cells from the oxidative damage caused by reactive oxygen species/reactive nitrogen species. Recent studies demonstrate that low dimensional carbon antioxidative nanomaterials have received a lot of attention owing to their tiny nanoscales and unique physicochemical property. As a result, a brief overview of recent advancements in antioxidant low-dimensional carbon materials is provided. Typically, carbon nanomaterials are classified according to their nanostructure dimensions, which are zero-dimension, one-dimension, and two-dimension. Last but not least, the challenges and perspectives of these high-performance low-dimensional materials in biomedical fields and further clinical usages are discussed as well.

## Introduction

The discovery of reactive oxygen species (ROS) originated from the study of oxidative damage. Oxygen is partially reduced to oxidizing free radicals through respiratory chain reactions, which are named “reactive oxygen species.” Typical ROS contains hydroxyl radical (·OH), superoxide radical anion (O_2_^•-^), singlet oxygen (^1^O_2_), and hydrogen peroxide (H_2_O_2_). Represented by nitric oxide (NO), reactive nitrogen species (RNS) is another oxide that plays an important role in the metabolism of living organisms. Typical RNS including NO radical (NO·), nitroso ion (NO^+^), peroxynitrite (ONOO^−^), S-nitrosomercaptan (SNOs), and nitrogen oxides (NO_x_) can be derived from the interaction between NO and various ROS. Moreover, ROS could convert into RNS in the presence of enzyme in cells (Liu and Shi, 2019). ROS and RNS act as essential roles in the body, participating in normal cell metabolism and responding to external stimuli by mediating signal transduction. The electrons carried by ROS and RNS grant them sensitive reactivity and can oxidize a variety of molecules including DNA, lipid, and proteins. As a double-edged sword, oxidation facilitates the defense system of living things to fight against invaders like bacteria, but ROS and RNS will cause damage to the body if they are out of control. Normally, the generation and removal of ROS and RNS are in a delicate dynamic balance. The cellular antioxidant system is responsible for removing excessive ROS and RNS. Once the balance is broken, whether excessive ROS and RNS are produced in the cell or the antioxidant system of the cell fails, oxidative stress occurs, marked by the oxidation of a variety of molecules and the following inflammatory reactions. Numerous studies have confirmed the causal relationship between oxidative stress and various diseases, such as inflammation, cardiovascular disease, autoimmune disorders, and neurodegenerative diseases. Therefore, antioxidant therapy is expected to prevent and treat oxidative stress-related diseases.

As a class of substances that can scavenge free radicals, antioxidants are usually formed within the body or outside the body. The endogenous antioxidants are mainly various natural enzymes or small molecules. Superoxide dismutase (SOD) and catalase (CAT) can catalyze the conversion of O_2_^·-^ into water and molecular O_2_, whereas glutathione peroxidase (GPx) can convert H_2_O_2_ and fatty acid hydroperoxides. But the substrate specificity and pH sensitivity of enzymes limit their ability to eliminate ROS and RNS in practical application. As a supplement, endogenous macromolecules (bilirubin, albumin) and other small biomolecules (vitamins, cysteine) have been applied to prevent oxidative damage. The disadvantage of these molecules is that they are less safe and may cause a series of clinical symptoms, including but not limited to bleeding, hemorrhagic stroke, and cancer incidence. The shortcomings of endogenous antioxidants prompted researchers to develop exogenous antioxidants. Recently, nanomaterials have drawn the attention of scientists due to their various advantages. On the one hand, nanomaterials can change the pharmacokinetics of natural antioxidant molecules when they act as carriers. Moreover, nanomaterials with the activities of antioxidative enzymes usually exhibit higher stability and a stronger tolerance to harsh microenvironments. In view of further clinical translation, these antioxidative nanomaterials seem much more feasible and attract great attention.

Several kinds of antioxidative nanomaterials have been well identified including metal-based nanomaterials, bioinspired polymer-based nanomaterials, and carbon-based nanomaterials. As typical bioinspired polymer-based nanomaterials, polydopamine (PDA) nanoparticles with excellent antioxidative activity could act as efficient scavengers for ROS and RNS in the treatments of periodontal disease and ischemic stroke (Liu et al., 2016; Bao et al., 2018). Moreover, various novel metal-based nanomaterials, such as Mn_3_O_4_ and Pt-modified PCN222-Mn MOFs, exhibited admirable ROS/RNS removal efficacy *in vivo*, which could protect live mice from both ear-inflammation and inflammatory bowel disease (Yao et al., 2018; Liu et al., 2020). Significantly, low-dimensional carbon-based nanomaterials have been proved to hold multiple radical scavenging abilities and exceptional antioxidative stability (Sun et al., 2018; Liu and Shi, 2019). Furthermore, advantages like low cost and ease of operation make these nanomaterials more attractive in further applications. In this review, we summarize the antioxidant properties of low-dimensional carbon materials based on their fascinating structures. We also highlight the potential antioxidant mechanism and recent biomedical usages of these low-dimensional carbon nanomaterials. Finally, we discuss the challenges and opportunities presented by low-dimensional carbon materials from the viewpoint of further clinical translation.

## Application of antioxidant low-dimensional carbon materials in biomedical fields

A wide range of carbon-based nanomaterials with antioxidative properties have been well explored including carbon particles, carbon sheet structure, carbon nanotubes, carbon clusters, and carbon dots. Here, we classified the carbon-based nanomaterials according to their structure as zero-dimension, one-dimension, two-dimension, and three-dimension. Moreover, we discuss the practical usage of low-dimensional carbon materials against numbers of ROS in biomedical fields because of their special structural properties and relatively high antioxidant activity.

### Zero-dimension carbon nanomaterials

**FIGURE 1:**
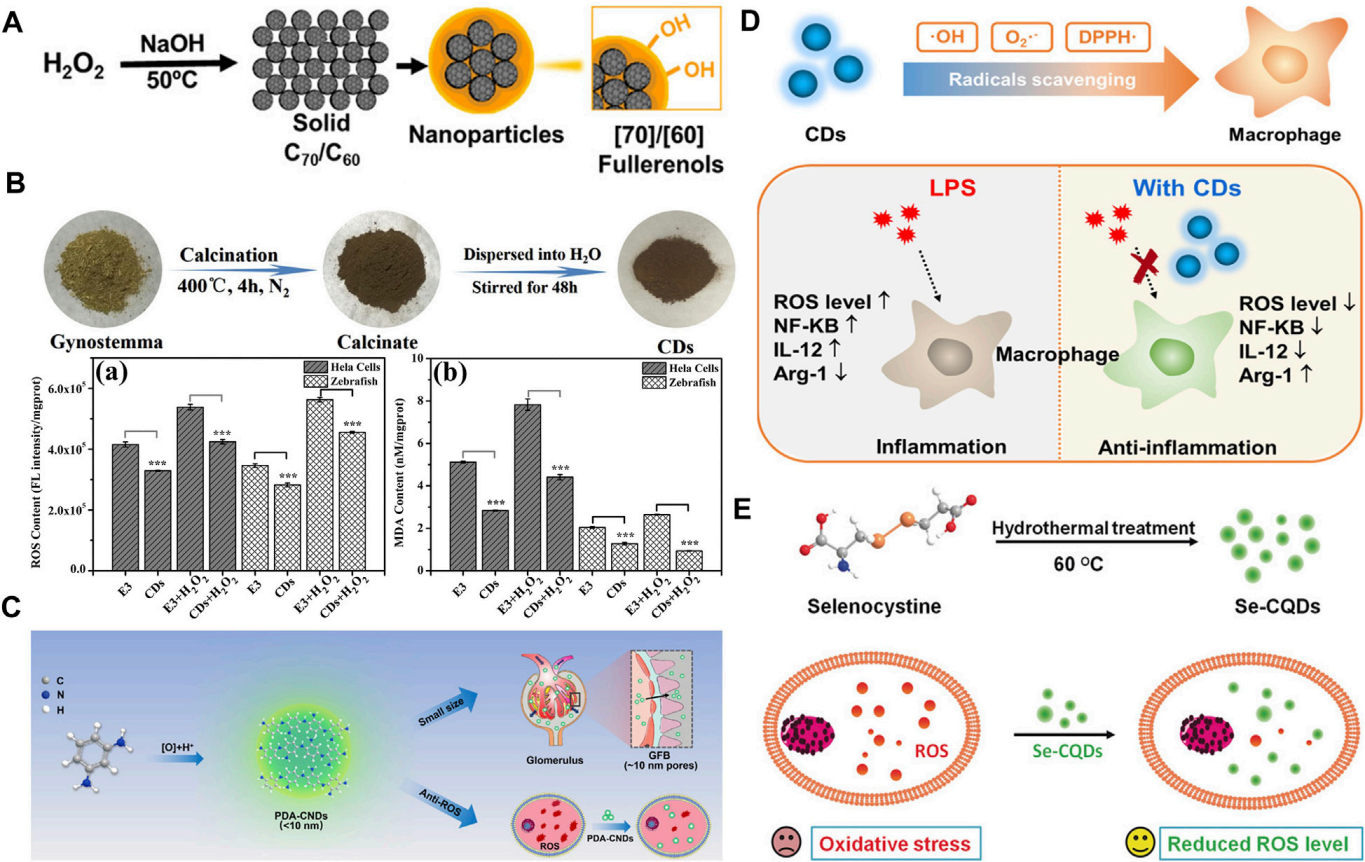
**(A)** Preparation of C_70_-OH and C_60_-OH nanoparticles against oxidative injury (Zhou et al., 2017). **(B)** Carbon dots derived from gynostemma as antioxidant against H_2_O_2_-induced oxidative in both Hela cells and zebrafish (Wei et al., 2019). **(C)** Phenylenediamine-based carbon nanodots were used to eliminate ROS in acute kidney injury (Gao et al., 2020). **(D)** Carbon dots made of citric acid and glutathione serve as a highly efficient intracellular ROS scavenger in macrophage (Wang et al., 2020a). **(E)** Selenium-doped carbon quantum dots protecting biosystems from oxidative stress (Li F. et al., 2017).

Since the first report of fullerene as an antioxidant, various fullerenes has been well investigated for its unique cage structure and scavenging activities towards OH and O_2_^·-^. The conjugated double bonds and the low-lying lowest unoccupied molecular orbital can account for their excellent antioxidative ability. Though they bears the name of “Radical Sponge,” their practical biomedical usages are often limited by the hydrophobic nature of original fullerenes. Derivatives including fullerenols, carboxyl-modified fullerenes, metal-modified fullerenes, and ethylenediamine-modified fullerenes usually exhibit better water solubility. Recent studies indicate that fullerenols can well eliminate free radicals both *in vivo* and *in vitro*. Better biocompatibility of fullerenol than native fullerene can be explained by its excretion in urine. So the fullerenol can be used to protect tissues or organs against oxidative injury induced by doxorubicin, Co. γ-rays and reduplicative chemotherapy (Figure 1A) (Zhou et al., 2017). The cage size, in particular, will affect the radical scavenging capability indicated by C_70_ fullerenols’ superior protection over C_60_-fullerenols. The reason for the difference can be attributed to the fact that C_70_-fullerenols hold more intrinsically coupled double bonds, is closer to electrons, and is more polarizable.

Carboxyl modification is another strategy for hydrophilic alternation. As a high-performance neuroprotective antioxidant, C_60_(C(COOH)_2_)_3_ has been shown to effectively prevent dopaminergic neuron apoptosis *in vitro* and protect cerebellar granule cells from apoptosis. Then, C_60_(C(COOH)_2_)_2_ dots with similar structure were well synthesized and tested for their ability to protect the blood-brain barrier. As expected, C_60_(C(COOH)_2_)_2_ dots can enter oxidized cerebral microvessel endothelial cells (CMECs) rather than normal cells and maintain CMEC integrity by inhibiting H_2_O_2_-induced F-actin depolymerization. It is of interest that carboxyl and cage size have an influence on the level of oxidative stress relaxation. To answer this question, different origins of acid including dimalonic acid, trimalonic acid, and quadrimalonic acid are selected and used to react with C_60_ and C_70_, respectively. These results suggest dimalonic acid-modified C_70_ fullerenes and trimalonic acid-modified C_70_ fullerenes exhibit an obviously more protective effect than others. The authors consider that the extended system and lower symmetric structure of the C_70_ fullerene cage, which possess higher activated electron-deficient areas on the cage surface, can extremely quench various free radicals (Liu et al., 2013).

The typical representative of the metallofullerene family is Gd@C_82_, while gadolinium endohedral metallofullerenol comprises a functionalized C_82_ fullerene cage with metal trapped inside (Yin et al., 2009). It has been reported that the chemical and physical properties of gadolinium endohedral metallofullerenols are determined by the number and position of the hydroxyl groups on the fullerene cage. These metallofullerenols hold excellent scavenging activity against reactive oxygen species and exhibit great cell protection effect. Amino acid modification is also used to change the hydrophilic properties; Zhou et al. find that L-lysine modified C_70_ fullerenes reveal higher scavenging activity than L-alanine modified C_70_ fullerene (Zhou et al., 2018). The difference is because of the different integrity of fullerene cage. Recently, C_60_-PDA-GSH are rationally designed and synthesized, and their relative cytoprotective effects against oxidative stress provide a candidate for the treatment of ROS-related diseases (Zhang et al., 2019). During the typical synthesis, C_60_ is modified by the self-polymerization of dopamine into C_60_-PDA hybrids. Subsequently, glutathione (GSH) is covalently immobilized onto the surface of C_60_-PDA hybrids *via* Michael addition reaction. Inspired by above successful preparation of various derivatives and relative modifications, Chen and her colleagues compare the discrepancy of three fullerene derivatives on free radical scavenging efficiency (Yin et al., 2009). C_60_[C(COOH)_2_]_2_ are found to be significantly less active than carboxyl-free fullerenes C_60_(OH)_22_ and Gd@C_82_(OH)_22_. Of particular importance, these fullerene derivatives can neutralize all physiologically relevant ROS and RNS, promising great potential in biomedical applications.

Generally, carbon dots (CDs) usually have an average particle size of 2–8 nm, and they have been widely used in biomedical fields due to their good photochemical properties, biocompatibility, as well as stability. Besides the bioimaging applications based on the fascinating fluorescence performance, some CDs have also been employed to treat oxidative stress-related diseases because of their great ROS scavenging ability. For instance, CDs prepared by using gynostemma and garlic as carbon source have been successfully synthesized (Figure 1B) (Wei et al., 2019). These CDs can efficiently eliminate excess ROS in both Hela cells and zebrafish. In another study, phenylenediamine-based CDs have been well designed and constructed for the treatment of acute kidney injury. The ultra-small size can facilitate the CDs to penetrate the glomerular filtration barrier and present the antioxidant properties at the kidney target (Figure 1C) (Gao et al., 2020). Since the various origins of carbon have been reported, it has provided an excellent demonstration for the “bottom-to-top” strategy. However, the mechanism of the antioxidant effect is rarely elucidated. Besides the precursor selection method mentioned above, some agents are used to enhance the antioxidant ability. A kind of CDs made from glutathione and citric acid *via* hydrothermal method has been designed with the ability to eliminate various free radicals including DPPH· (1,1-diphenyl-2-trinitro phenylhydrazine free radicals, a kind of experimental RNS), ·OH, and O_2_·^-^ (Figure 1D) (Wang et al., 2020a). In addition, metallic heteroatom selenium has been doped into the CDs to fulfill the need for OH scavenging and imaging at the same time (Figure 1E) (Li F. et al., 2017).

**FIGURE 2:**
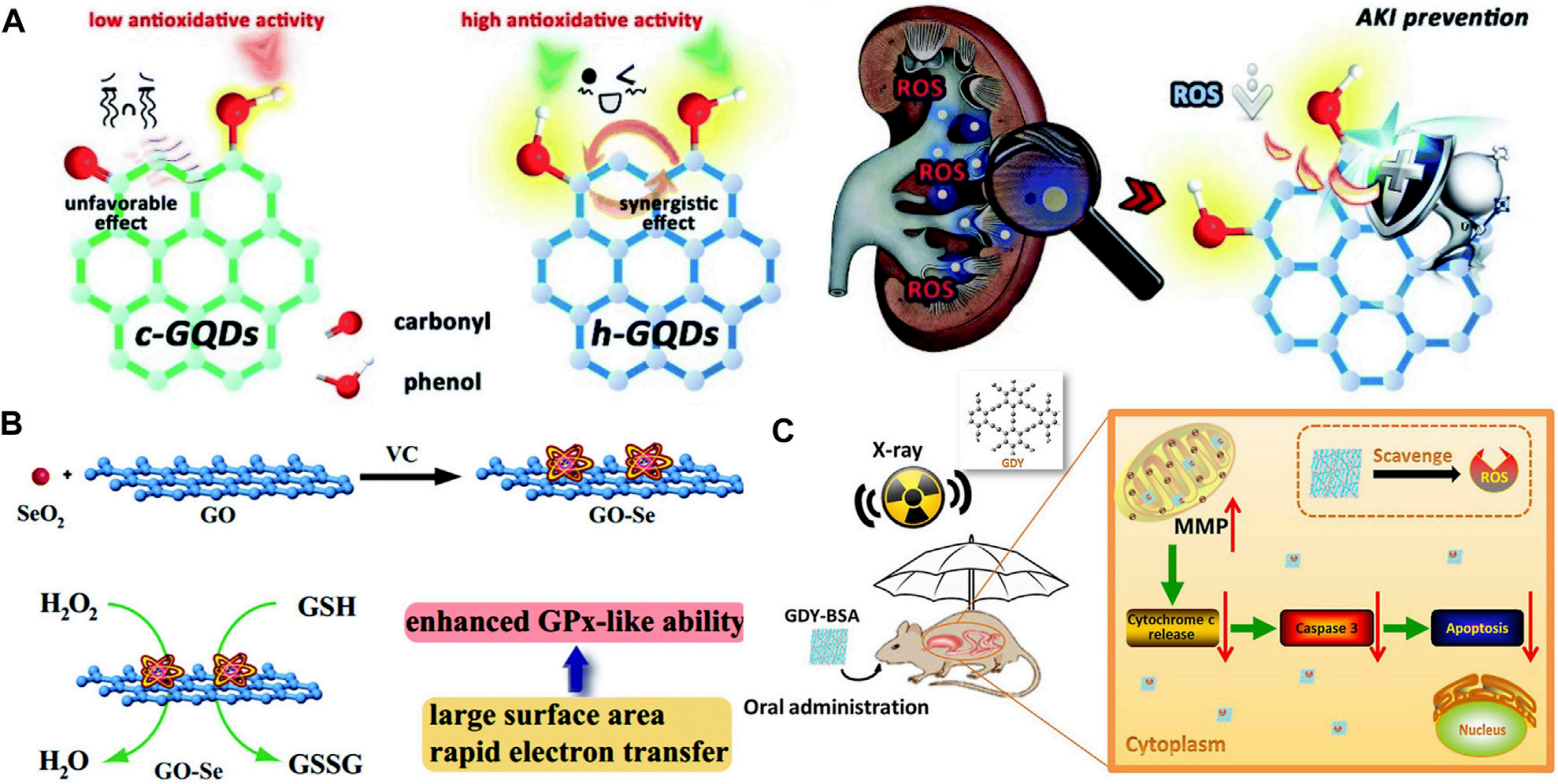
**(A)** Usage of phenol-like group functionalized graphene quantum dots (h-GQDs) for the treatment of acute kidney injury (Wang et al., 2020b). **(B)** GO-Se nanocomposites with great GPx-like properties to alleviate cellular oxidative stress (Huang et al., 2017). **(C)** Oral administration of BSA-modified GDYs for gastrointestinal radioprotection (Xie et al., 2020).

As a typical representative of CDs, graphene quantum dots (GQDs) are well researched because of their low toxicity and unique structure. Researchers have created GQDs with antioxidant characteristics, which can efficiently scavenge free radicals and protect cells from oxidative damage. They say ROS was eliminated *via* surface imperfections and unpaired electrons. Furthermore, the *π*-conjugated nature facilitated electron transformation and storage (Chong et al., 2016). Based on this theory, phenol-like group functionalized GQDs were rationally designed and well proved to be a better antioxidant in the acute kidney injury model (Figure 2A) (Wang et al., 2020b). Other attempts to improve scavenging results have been made, such as the use of phosphorus-doped GQDs in the efficient elimination of DPPH and ·OH (Li Y. et al., 2017).

### One-dimension carbon nanotubes

Carbon nanotubes (CNTs) are seamless cylinders of rolled-up graphene sheets with distinct intrinsic characteristics, which can be divided into single-wall and multiwall carbon nanotubes, with diameters ranging from 0.4 to 2.5 nm and a few nanometers to 100 nm, respectively. CNTs are believed to be a radical scavenger due to their exceptional electron donation and acceptability abilities acquired from conjugated carbon double bonds. Researchers demonstrate that pristine single-walled CNTs and phenolic-functionalized CNTs are powerful antioxidants (Lucente-Schultz et al., 2009). According to another study, two types of carbon nanotubes with varying thicknesses and lengths exhibit comparable free radical scavenging efficacy. CNTs with smaller diameters and shorter lengths have been shown to be more poisonous and capable of causing inflammation. In another study, the effects of carbon nanotube length, diameter, and chirality on their ability to scavenge free radicals are calculated theoretically (Galano, 2009). According to previous studies, the length and chirality of CNTs usually reveal no effect on antioxidant efficacy. The antioxidant activity of armchair nanotubes can be more affected by changes in tube diameter. Aside from physical properties, both amine modification and polydopamine modification can improve the antioxidant activities of CNTs, as demonstrated in stroke and skin models (Lee et al., 2011; Liang et al., 2019). The location of defects in the material also influences their ability to scavenge free radicals. Methods like ball milling, benzoyl peroxide, and acid treatment can consecutively create defect sites, increasing free radical scavenging ability, whereas microwave treatment repair flaws. Significantly, extended microwave time can extremely reduce fault locations and free radical capabilities (Shieh and Wang, 2014).

### Two-dimension carbon graphene and graphdiyne

Since the first discovery of graphene (G) in 2004, a number of its derivatives, from graphene oxide (GO) to reduced graphene oxide (RGO), have been well investigated in various fields. The obvious difference between G and GO is the addition of oxygen atoms bound to the carbon scaffold. As a result, G is hydrophobic in nature, whereas GO is hydrophilic, which is more applicable *in vivo*. RGO is reported to react with DPPH·, O_2_^·-^, and OH. The ability to clear OH has been ranked as high as few-layer graphene, GO, and RGO. These results indicate that the primary active sites are highly associated with the pristine graphenic network rather than oxygen-containing functional groups (Qiu et al., 2014; Suresh et al., 2015). In addition, the oxidation resistance of G is also solved through its use as a template to enhance the oxidation activity of molecules of clusters. Huang et al. have reported that GO-Se nanocomposites with excellent GPx-like properties can efficiently protect cells against oxidative stress *in vitro* (Figure 2B) (Huang et al., 2017).

As a new 2D form of carbon, graphdiyne (GDY) is only one atom thick. Owing to the presence of its good sp- and sp^2^-hybridized carbon atoms, evenly spaced pores, high conjugation, and unique electronic, physical, and chemical properties, a lot of work has been done to find ways to use it. It is a 2D periodic structure whose fundamental structural unit is a big triangular ring containing 18 carbon atoms. Due to the unique structure of GDY, it has been proved that GDY usually exhibits better performance and higher stability than other carbon-based materials in various practical applications including the fields of energy and biomedicine. For the scavenging free radicals usages, bovine serum albumin (BSA)-modified GDYs have been used as a high-performance radioprotector (Figure 2C) (Xie et al., 2020). These BSA-modified GDYs hold a strong free radical scavenging ability including O_2_^·-^ and OH. By eliminating intracellular ROS, BSA-modified GDYs can extremely reduce DNA damage and protect major organs from radiation-induced damage *in vivo*. In addition, GDYs can be doped into sodium hyaluronate hydrogel to form nano-graphdiyne hydrogels (Xie et al., 2022). These well-prepared graphdiyne-contained hydrogels not only exhibit great broad-spectrum free radical scavenging activity, but also process admirable physically shielding function against low-energy X-ray, which can be used for the external skin radioprotection. Compared to G and its derivatives, GDYs and their derivatives seem to hold better free radical scavenging activity in biomedical fields, which can be researched more in the future.

## Conclusion and perspective

In this review, we summarize the rational design and synthesis of 0-, 1-, and 2-dimensional carbon materials and their applicability as antioxidants in biomedical fields. As expected, low-dimensional carbon materials can offer the benefits of a high ratio of surface area to volume, ease of modification, and high-efficiency electron transmission, as well as promising anti-oxidation regulation and application potential. Prior to further clinical translation, numbers of concerns must be further researched and resolved. First, as dual-active materials, carbon-based nanomaterials can not only scavenge ROS/RNS, but also demonstrate a promotion effect on ROS/RNS formation in some settings; however, the mechanism underlying this action remains unknown. Accordingly, the impact of this dual activity on biological use must be taken into account in order to determine the optimal therapeutic dose and typical application environment. Second, the ultra-small size and strong reactivity of low-dimensional carbon materials necessitate the additional research into their biosafety and systemic toxicity. Future studies must focus on the long-term toxicity and multiple organ toxicity after proper administration, given that the vast majority of clinical application situations require materials to reach the bloodstream. How to evaluate the relationship between the structure and oxidation resistance of low-dimensional carbon materials and how to realize their relative anti-oxidative properties by selecting appropriate preparation strategy, surface modification, element doping, as well as other factors must be the final and most fundamental issue.
